# Supplementary material for: Mechanism of dimer selectivity and binding cooperativity of BRAF inhibitors
Source: eLife. 2025 Feb 13;13:RP95334. doi: 10.7554/eLife.95334 (PMC11825127; doi:10.7554/eLife.95334)
Supplement: Supplementary file 1. — Table A . List of the BRAFV600E inhibitors and the structure features of the co-crystal structures in the PDB. The monomer and dimer selectivities of inhibitors in black are based on the experimental data in Adamopoulos et al., 2021 and Cotto-Rios et al., 2020 (PHI1). The monomer and dimer selectivities of inhibitors in red were predicted by us and supported by experiments in Karoulia et al., 2017 (GDC0879) and Tkacik et al., 2023 (Tovorafenib). Note, the PDB entries indicated by an astrisk are co-crystal structures in complex with the wild type BRAF (the BRAFV600E forms are unavailable). All structures contain an inhibitor in each protomer, with the exception of PLX7904; in the PDB entry 4XV1, PLX7904 is only present in protomer A. The C helix position (in Å) is defined in the main text. Two values refer to the two protomers. The back pockets (BPs) occupied was calculated by KLIFS (Kooistra et al., 2016) based on the definition of Liao, 2007. K-E refers to the distance (in Å) between the amine nitrogen of Lys483 and the nearest carboxylate oxygen of Glu501. Table B: Average value and standard deviation of reported quantities from out dimeric BRAF simulations, separated by system and protomer. Each quantity was calculated for each replica after removing the first 2 s for equilibration. [file elife-95334-supp1.docx]

**Supplemental Table A**

| Inhibitor | PDB | ⍺C;DFG | BPs | BP H-bonds | ⍺C position | K-E |
| --- | --- | --- | --- | --- | --- | --- |
| **Monomer selective** | | | | | | |
| Vemurafenib | 5JRQ | CODI | I,II | D594, G596 | 21.5/21.5 | 11.5/11.8 |
| Dabrafenib | 5CSW | CODI | I,II | K483, D594, G596 | 21.4/21.8 | 7.4/8.7 |
| PLX7904 | 4XV2 | CODI | I,II | D594, F595, G596 | 20.9/20.4 | 7.5/8.7 |
| **Equipotent** | | | | | | |
| AZ628 | 4G9R | CIDO | I,II,III | E501, D594 | 19.5/19.5 | 3.0/2.6 |
| TAK632 | 4KSP* | CIDO | I,II,III | E501, D594 | 18.8/19.0 | 3.0/3.9 |
| LY3009120 | 5C9C | CIDO | I,II,III | E501, D594 | 19.2/19.2 | 2.7/2.7 |
| Ponatinib | 6P3D | CIDO | I,II,III | E501, D594, I573, H574 | 19.2/19.2 | 3.0/3.0 |
| Lifirafenib  (BGB2893) | 4R5Y | CIDO | I,II,III | E501 | 19.0/18.7 | 5.5/5.1 |
| Tovorafenib (TAK580) | 6V34 | CIDO | I,II,III | E501, D594 | 18.6/18.8 | 2.5/2.9 |
| SB5909885 | 2FB8* | CIDI | I | E501 | 18.6/18.3 | 2.9/3.2 |
| GDC0879 | 4MNF | CIDI | I | E501 | 18.4/18.7 | 2.8/2.9 |
| **Dimer selective** | | | | | | |
| Naporafenib (LXH254) | 8F7P* | CIDO | I,II,III | E501, D594 | 19.3/19.3 | 3.0/3.0 |
| RAF709 | 5VAM* | CIDO | I,II,III | E501, D594 | 19.2/19.1 | 2.8/2.8 |
| Sorafenib | 1UWJ | CIDO | I,II,III | E501, D594 | 18.8/18.8 | 2.6/2.6 |
| Belvarafenib | 6XFP* | CIDO | I,II,III | E501, D594 | 19.3/19.3 | 2.7/2.7 |
| PHI1 | 6P7G | CIDO | I,II,III | E501, D594, H574 | 19.1/19.0 | 2.5/2.5 |

**Supplemental Table B**

| System, protomer | ⍺C position (Å) | K-E distance (Å) | DFG dihedral (°) |
| --- | --- | --- | --- |
| Apo monomer, A | 23.3 ± 1.7 | 8.1 ± 3.1 | 214 ± 19 |
| Apo dimer, A | 22.2 ± 1.0 | 6.5 ± 2.9 | 250 ± 39 |
| Apo dimer, B | 22.0 ± 1.7 | 5.5 ± 2.2 | 201 ± 24 |
| PHI1 (mixed), apo | 21.3 ± 1.0 | 5.5 ± 3.1 | 239 ± 41 |
| PHI1 (mixed), holo | 18.9 ± 0.7 | 2.8 ± 0.3 | 283 ± 24 |
| LY (mixed), apo | 22.8 ± 1.7 | 5.3 ± 2.4 | 204 ± 35 |
| LY (mixed), holo | 19.0 ± 0.5 | 4.9 ± 1.2 | 295 ± 13 |
| PHI1 (holo), A | 18.3 ± 0.6 | 2.8 ± 0.1 | 285 ± 17 |
| PHI1 (holo), B | 18.7 ± 0.7 | 2.8 ± 0.1 | 303 ± 12 |
| LY (holo), A | 19.4 ± 0.6 | 3.4 ± 0.1 | 301 ± 18 |
| LY (holo), B | 19.3 ± 0.5 | 4.3 ± 1.4 | 295 ± 13 |
